# Association between systemic inflammation and water composition and survival in colorectal cancer

**DOI:** 10.3389/fonc.2022.896160

**Published:** 2022-10-24

**Authors:** Shi-Qi Lin, Hai-Lun Xie, Yi-Zhong Ge, Guo-Tian Ruan, Qi Zhang, Meng-Meng Song, He-Yang Zhang, Xi Zhang, Xiang-Rui Li, Meng Tang, Xian Shen, Chun-Hua Song, Wei Li, Han-Ping Shi

**Affiliations:** ^1^ 1Department of Surgery, the Second Affiliated Hospital and Yuying Children's Hospital of Wenzhou Medical University, Wenzhou, China; ^2^ Department of Gastrointestinal Surgery, Beijing Shijitan Hospital, Capital Medical University, Beijing, China; ^3^ Department of Clinical Nutrition, Beijing Shijitan Hospital, Capital Medical University, Beijing, China; ^4^ Beijing International Science and Technology Cooperation Base for Cancer Metabolism and Nutrition, Beijing, China; ^5^ Key Laboratory of Cancer Food for Special Medical Purposes (FSMP) for State Market Regulation, Beijing, China; ^6^ Department of Cancer Center, the First Hospital of Jilin University, Changchun, Jilin, China; ^7^ Cancer Center of the First Hospital of Jilin University, Changchun, China

**Keywords:** intracellular water/total body water ratio, neutrophil-to-lymphocyte ratio, colorectal cancer, inflammation-water score, inflammation

## Abstract

**Background:**

Systemic inflammation and water composition are important factors affecting cancer prognosis. This study aimed to explore the association between the neutrophil-to-lymphocyte ratio (NLR) and intracellular water/total body water (ICW/TBW) ratio and overall survival (OS) in colorectal cancer (CRC).

**Methods:**

This multicenter, prospective cohort included 628 patients with CRC between June 2012 and December 2019. The association between the covariates and OS was assessed using a Cox proportional hazards model and restricted cubic spline models. Concordance index (C-index), which integrated discriminant improvement (IDI) index and continuous net reclassification index, (cNRI) was used to compare the predictive ability of the markers.

**Results:**

The optimal cutoff values for the NLR and ICW/TBW ratio were 2.42 and 0.61, respectively. The NLR was negatively associated with OS, while the ICW/TBW ratio was positively correlated with OS. NLR ≥2.42 and ICW/TBW ratio <0.61 were both independent poor prognostic factors (hazard ratio [HR]: 2.04, 95% confidence interval [CI]: 1.44–2.88 and HR: 1.45, 95% CI: 1.04–2.02, respectively). Subsequently, we combined the two factors to construct an inflammation-water score (IWS). Patients with IWS (2, ≥1) had worse OS (HR: 2.86 and 95% CI: 1.77–4.63; HR: 1.74 and 95% CI 1.17–2.57, respectively) than those without one. Compared to its component factors, IWS score showed better predictive ability for C-index, IDI index, and cNRI.

**Conclusion:**

A high NLR and a low ICW/TBW ratio were independent risk factors for poor prognosis in patients with CRC. The combination of the two factors can provide a better prognostic prediction effect.

## Introduction

Colorectal cancer (CRC) is the third most common cancer worldwide. Despite progress in screening and treatment, the incidence, prevalence, and mortality rates of CRC remain high. Approximately 1.9 million new cases of CRC and 935,000 related deaths were reported in 2020 wherein approximately one in ten cancer cases and deaths are due to CRC (1). Therefore, investigating appropriate markers to assess the prognosis of patients with CRC is necessary to achieve precise individualized treatments.

Systemic inflammation is one of the important factors affecting the occurrence and development of cancer. Systemic inflammation promotes cancer progression across all stages. First, inflammatory factors can directly promote tumor growth. In addition, it can affect the inflammatory tumor microenvironment and further affect tumor growth by promoting angiogenesis and inhibiting adaptive immune responses (2–4), among other mechanisms. As one of the most representative indicators of cancer-related inflammation, the prognostic value of neutrophil-to-lymphocyte ratio (NLR) has been demonstrated in various cancers, including colorectal (5–7), oropharyngeal (8), prostate (9) cancers.

In recent years, body water composition has also been reported as a useful predictor of cancer prognosis. Amano et al. (10) found that a high intracellular water (ICW) content was associated with a poor prognosis in patients with cancer-related edema (10). Hirashima and Noda et al. (11) reported that a high extracellular water (ECW)/total body water (TBW) ratio was associated with frailty and poor treatment tolerance in patients with lung cancer (11, 12). Due to the high water content of the human skeletal muscle, ICW is closely related to muscle mass. Cancer-related muscle loss and inflammation may lead to a decreased ICW and an intracellular-to-extracellular fluid transfer. According to Amano et al.’s (10) study, patients with advanced cancer with and without edema have a lower ICW than healthy controls while ECW evaluated. Further, Park et al. (13) found that in patients with sepsis, the ECW/TBW ratio of non-survival increased with the decrease of the ICW/TBW ratio. As such, an intracellular-to-extracellular fluid transfer may take place in patients with a disease burden. Therefore, the fluid balance of the patients may be better evaluated using the ICW/TBW ratio. However, there is no report on the effects of the ICW/TBW ratio on cancer prognosis.

These two indicators are easy to obtain in the clinical environment and have a good predictive effect on cancer prognosis. The combination of the two may provide further reference for prognostic evaluation, curative effect evaluation, and treatment guidance for patients with cancer. To our knowledge, no studies have examined the association of systemic inflammation markers and water composition to prognosis in patients with CRC. Therefore, the purpose of this study was to investigate the relationship between NLR and ICW/TBW ratio and OS in patients with CRC.

## Methods and methods

### Study population

The patients in this study were from the Nutritional Status and Clinical Outcomes Survey of Common Cancers in China project (registration number: ChiCTR1800020329). The detailed design and recruitment and exclusion criteria of the Nutritional Status and Clinical Outcomes Survey of Common Cancers in China project have been reported in the previous literature (14). Briefly, this cohort recruited cancer patients over 18 years of age in more than forty centers across China, between June 2012 and December 31, 2019, with the primary objective of exploring the relationship between nutritional status and clinical outcomes. In this article, we screened patients with pathologically diagnosed colorectal cancer and mainly explored the relationship between ICW/TBW, NLR and survival outcomes. The primary outcome of this study was patient overall survival. The exclusion criteria comprised the absence of the following data: water composition, neutrophil count, lymphocyte count, age, and tumor-node-metastasis (TNM) stage (**Figure S1**). This study was approved by the institutional review committees of all the participating agencies. All the participants provided written informed consent.

### Data collection

The clinicopathological data collected from the patients’ electronic medical records were as follows: demographic characteristics (age, sex, and BMI), lifestyle (smoking and alcohol history), TNM stage, and treatments (surgery, chemotherapy, and radiotherapy). Water components, including ICW and TBW, were measured through direct segmental multi-frequency bioelectrical impedance analysis (InBody S10 Body Water Analyzer; InBody). The Inbody S10 requires subjects to perform the test in a fasted, resting state to ensure redistribution of water in the body. This instrument is connected to the limbs through a plurality of contact electrodes, and sends out a weak current to detect the electrical impedance of the human body to obtain relevant data such as phase angle, FFM, FM, ICW and TBW (13, 15). Serological testing included neutrophil, lymphocyte counts, total protein, albumin and globulin, which were collected 10 hours after fasting (before treatments). Baseline data including ICW, TBW, FFM (fat free mass), FM (fat mass) and serological testing were obtained within 48 hours of admission. All measurements were standardized to explain the measurement errors between laboratories. TNM stage was defined according to the 8th American Joint Committee on Cancer TNM classification system. The neutrophil count was divided by the lymphocyte count to calculate the NLR. The ICW was divided by the TBW to calculate the ICW/TBW ratio.

### Statistical analyses

The continuous variables of skewness distribution are presented as medians (quartile range) and were analyzed using the Kruskal–Wallis test. The categorical variables are presented as frequency (percentage) and were analyzed using the Pearson χ2 test. Restricted cubic splines were established to evaluate the nonlinear relationship between the NLR and ICW/TBW ratio and the OS. The cutoff value of the NLR and ICW/TBW ratio were calculated using a standardized log-rank statistic (survminer R package). A linear model was used to evaluate the correlation between the NLR and ICW/TBW ratio. The inflammation-water score (IWS) was established based on the NLR and ICW/TBW ratio. A Kaplan–Meier curve was used to draw survival curves, and a log-rank analysis was used for testing. After adjusting for confounding factors, univariate and multivariate Cox proportional risk models were used to assess the independent prognostic predictors of CRC. The concordance index (C-index), integrated discrimination improvement (IDI) index, and continuous net reclassification index (cNRI) were used to compare the predictive abilities of the NLR, ICW/TBW ratio, and IWS. The predictive abilities of the ICW/TBW, FFM, and FM were compared using time-dependent area under the curve (AUC) analysis. Statistical significance was set at *P <*0.05, and R software (version 4.1.2) was used for data analysis.

## Result

### Baseline characteristics of population

Of this study of 628 subjects, 376 (59.9%) were male and the median age was 60 years. Of these, 36 (5.7%) patients were in TMN stage I, 133 (21.2%) in TMN stage II, 257 (40.9%) in TMN stage III, and 202 (32.2%) in TMN stage IV. The median follow-up in this study was 22.33 months and a total of 171 (27.3%) people died during the follow-up period (**Table 1**).

**Table 1:** Baseline characteristics of patients with colorectal cancer stratified by NLR and ICW/TBW ratio.

Characteristics	Overall N = 628	NLR			ICW/TBW ratio		
		Low	High	*P*	Low	High	*P*
		N = 339	N = 289		N = 260	N = 368	
Sex [male, n (%)]	376 [59.87]	185 [54.57]	191 [66.09]	0.004	135 [51.92]	241 [65.49]	0.001
Age [median (IQR)]	60.00 [13.00]	58.00 [12.00]	62.00 [13.00]	<0.001	63.00 [12.00]	57.00 [14.00]	<0.001
BMI [median (IQR)]	22.85 [4.64]	22.58 [4.36]	23.11 [4.84]	0.356	22.06 [4.20]	23.35 [4.88]	<0.001
Smoking [yes, n 9%)]	239 [38.06]	121 [35.69]	118 [40.83]	0.215	91 [35.00]	148 [40.22]	0.214
Drinking [yes, n (%)]	126 [20.06]	65 [19.17]	61 [21.11]	0.615	42 [16.15]	84 [22.83]	0.051
Surgery [yes, n (%)]	167 [26.59]	38 [11.21]	129 [44.64]	<0.001	68 [26.15]	99 [26.90]	0.907
Chemoradiotherapy [yes, n (%)]	379 [60.35]	257 [75.81]	122 [42.21]	<0.001	160 [61.54]	219 [59.51]	0.668
Tumor stage [n (%)]				0.029			0.188
I	36 [5.73]	15 [4.42]	21 [7.27]		13 [5.00]	23 [6.25]	
II	133 [21.18]	65 [19.17]	68 [23.53]		46 [17.69]	87 [23.64]	
III	257 [40.92]	156 [46.02]	101 [34.95]		108 [41.54]	149 [40.49]	
IV	202 [32.17]	103 [30.38]	99 [34.26]		93 [35.77]	109 [29.62]	
Neutrophil [median (IQR)]	3.26 [2.61]	2.40 [1.35]	4.89 [3.35]	<0.001	3.36 [2.90]	3.19 [2.50]	0.575
Lymphocyte [median (IQR)]	1.48 [0.75]	1.70 [0.69]	1.19 [0.69]	<0.001	1.44 [0.71]	1.50 [0.81]	0.539
ICW [median [IQR)]	21.20 [6.40]	20.50 [6.70]	21.90 [6.00]	0.034	19.50 [5.45]	22.70 [6.10]	<0.001
TBW [median (IQR)]	35.00 [10.22]	33.80 [10.80]	35.80 [9.30]	0.033	32.50 [9.40]	36.65 [9.95]	<0.001
NLR [median (IQR)]	2.25 [2.61]	1.45 [0.74]	4.21 [3.52]	<0.001	2.32 [2.69]	2.24 [2.50]	0.44
ICW/TBW ratio [median (IQR)]	0.61 [0.01]	0.61 [0.01]	0.61 [0.02]	0.158	0.60 [0.01]	0.62 [0.01]	<0.001
Status [death, n 9%)]	171 [27.23]	62 [18.29]	109 [37.72]	<0.001	88 [33.85]	83 [22.55]	0.002

BMI, body mass index; NLR, neutrophil-to-lymphocyte ratio; ICW, intracellular water; TBW, total body water; ICW/TBW ratio, intracellular water/total body water ratio.

### Association between the NLR and survival

**Figure 1:**
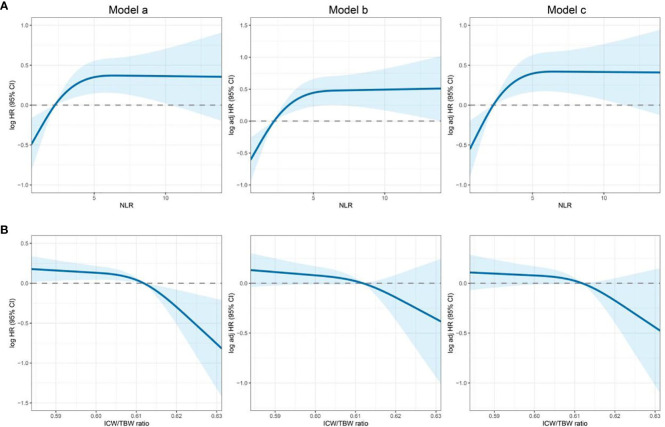
Association of the NLR and ICW/TBW ratio to all-cause mortality in patients with colorectal cancer. **(A)**- NLR, neutrophil-lymphocyte ratio; **(B)**- ICW/TBW ratio, intracellular water/total body water ratio. Model a: Crude model, Model b: Adjusted for age, sex, BMI, and TNM stage, Model c: Adjusted for age, sex, BMI, TNM stage, smoking, drinking, surgery, and chemoradiotherapy.

**Figure 2:**
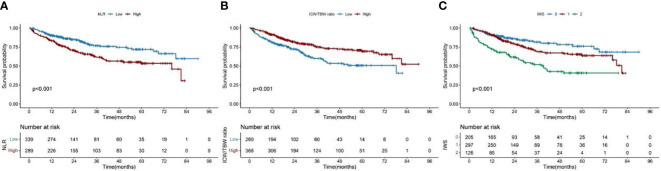
Kaplan–Meier curve of the NLR and ICW/TBW ratio, and inflammation-water score in patients with colorectal cancer. **(A)**- NLR, neutrophil-lymphocyte ratio; **(B)**- ICW/TBW ratio, intracellular water/total body water ratio; **(C)**- IWS, inflammation-water ratio.

The cutoff value of the NLR was 2.42. According to this value, 289 (46.02%) and 339 (53.98%) patients were classified into the high and low NLR groups, respectively. A high NLR was significantly associated with males, advanced ages, high neutrophil counts, low lymphocyte counts, and high all-cause mortalities (**Table 1**). The restricted cubic spline models showed that the NLR had an inverted L-shaped relationship with the OS of patients with CRC (**Figure 1A**). Compared with that of NLR <2.42, the hazard ratio (HR) (95% confidence interval [CI]) of the all-cause mortality in patients with NLR ≥2.42 was 2.00 (1.42–2.81) (**Table S2**). Upon dividing the NLR into quartiles, both Q3 and Q4 were found to be positively correlated with poor OS (*P* for the trend = 0.001). The HR (95% CI) of the all-cause mortality for Q4 was 1.83 (1.14–2.94). The Kaplan–Meier curve showed that the mortality of the high NLR group was higher than that of the low NLR group (**Figure 2A**). The results of a stratified analysis showed that a high NLR was consistently associated with an increased risk of mortality in almost all the subgroups of patients with CRC (**Figure S5A**).

### Association between the ICW/TBW ratio and survival

In **Figure S2**, we found that the predicted viability of ICW/TBW was higher than that of FM and FFM, so in this study, we chose ICW/TBW for further analysis. The cutoff value of the ICW/TBW ratio was 0.61. According to this value, 260 (41.40%) and 368 (58.60%) patients were classified into the low and high ICW/TBW ratio groups, respectively. A low ICW/TBW ratio was significantly associated with females, advanced ages, low BMIs, no history of alcohol consumption, low ICWs, low TBWs, and high all-cause mortalities (**Table 1**). Generally, a negative correlation was observed between the ICW/TBW ratio and mortality (**Figure 1B**). After adjusting for all the confounding factors, we found that compared with that of the ICW/TBW ratio ≥0.61, the HR (95% CI) of the all-cause mortality in patients with ICW/TBW ratio <0.61 was 1.40 (1.01–1.95) (**Table S3**). Upon dividing the ICW/TBW ratio into quartiles, both Q1 and Q2 were positively correlated with worse prognosis (*P* for the trend = 0.048). The Kaplan–Meier curve showed that the prognosis of the low ICW/TBW ratio group was worse than that of the high ICW/TBW ratio group (**Figure 2B**). The results of the stratified analysis also showed that a low ICW/TBW ratio was associated with an increased risk of death in almost all the subgroups of patients with CRC (**Figure S5B**).

### Association between the NLR and ICW/TBW ratio

We further explored the linear association between NLR and ICW/TBW ratio, and the results showed that there was no significant correlation between the two (**Figure S3**). In addition, a high NLR (≥2.42) and low ICW/TBW ratio (<0.61) were independent predictors of poor survival in CRC (model c- HR: 2.04 and 95% CI 1.44–2.88; HR: 1.45 and 95% CI: 1.04–2.02, respectively; the NLR and ICW/TBW ratio were mutually adjusted) (**Table 2**).

**Table 2:** Cox regression analysis of ICW/TBW and NLR associated with overall survival.

Characteristics	Model a		Model b		Model c	
	HR (95% CI)	P	HR (95% CI)	P	HR (95% CI)	P
NLR						
<2.42	ref.		ref.		ref.	
≥2.42	1.92 (1.4, 2.63)	<0.001	2.16 (1.56, 2.99)	<0.001	2.04 (1.44, 2.88)	<0.001
ICW/TBW ratio						
≥0.61	ref.		ref.		ref.	
<0.61	1.86 (1.37, 2.51)	<0.001	1.48 (1.07, 2.05)	0.019	1.45 (1.04, 2.02)	0.028

NLR, neutrophil-to-lymphocyte ratio; ICW/TBW ratio, intracellular water-total body water ratio.

Model a: No adjusted.

Model b: Adjusted for age, sex, BMI, TNM stage.

Model c: Adjusted for age, sex, BMI, TNM stage, smoking, drinking, surgery, chemoradiotherapy.

In three models ICW/TBW ratio (≥0.61, <0.61) and NLR (<2.42, ≥2.42) were adjusted mutually.

### Association between the IWS and survival

**Figure 3:**
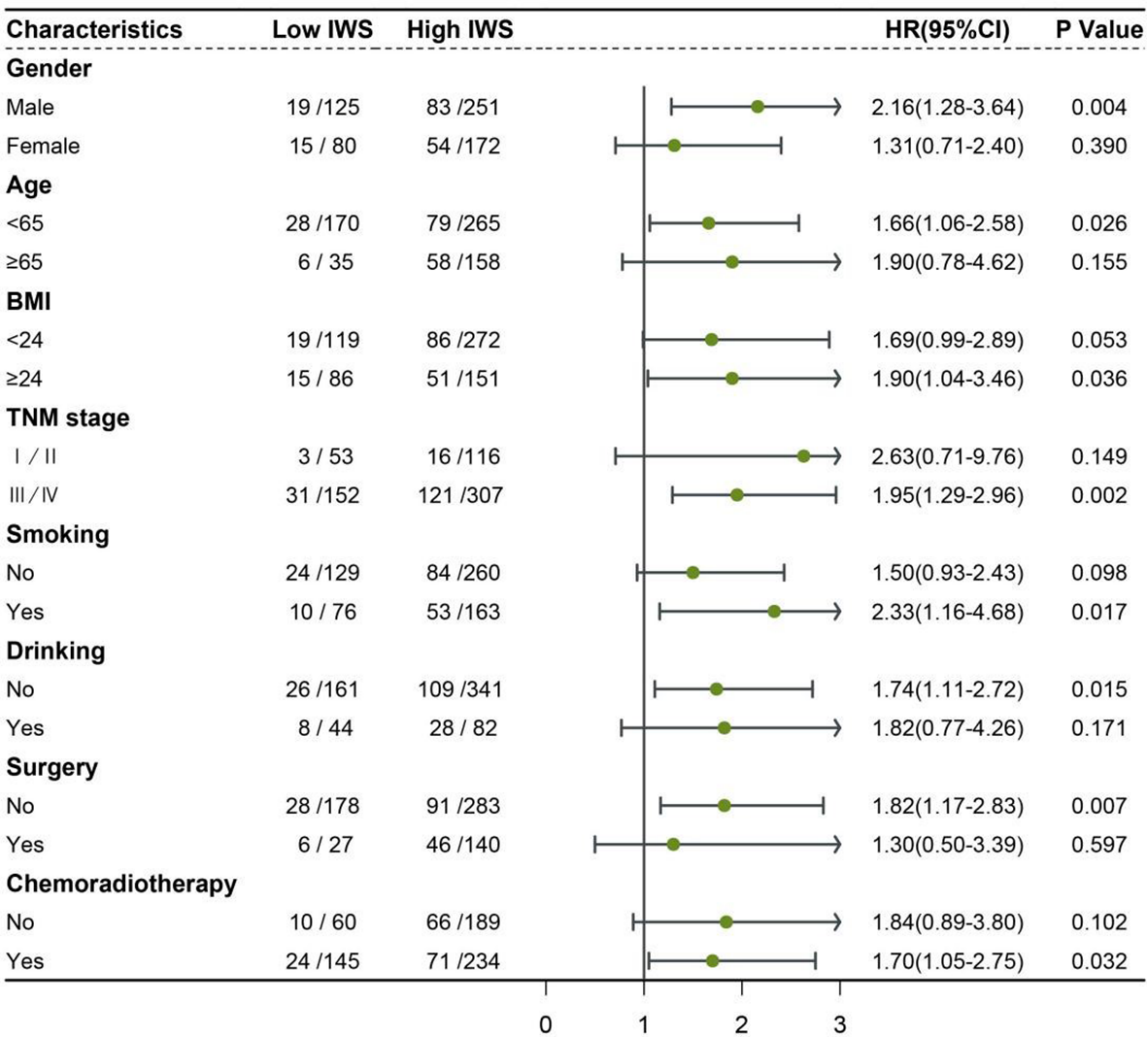
Association between the inflammation-water score and hazard risk of overall survival in various subgroups IWS: inflammation-water score (low IWS = 0 and high IWS ≥1) Adjusted for age, sex, BMI, TNM stage, smoking, drinking, surgery, and chemoradiotherapy.

The IWS was established based on the cutoff values of the NLR and ICW/TBW ratio: 0 point for NLR <2.42 and ICW/TBW ratio ≥0.61, 1 point for NLR ≥2.42 or ICW/TBW ratio <0.61, and 2 points for NLR ≥2.42 and ICW/TBW ratio <0.61 (**Table 3**). The Kaplan–Meier curve of the total population showed that patients with an IWS of 0 had the best survival, and patients with an IWS of 2 had the worst survival (*P <*0.001, **Figure 2**). Based on different TNM stages, there was a significant difference between the IWS and OS in all stages. The multivariate Cox regression model showed an association between the two (**Table 4**). In model c, compared with that of an IWS of 0, the HRs (95% CI) of the all-cause mortality in patients with an IWS of 1 and 2 were 1.49 (0.98–2.24) and 2.86 (1.77–4.63), respectively, and the HR (95% CI) of the all-cause mortality in patients with an IWS of ≥1 was 1.74 (1.17–2.57). The variate subgroups showed that patients with a high IWS (≥1) had a significantly higher all-cause mortality than those with a low IWS (=0) (**Figure 3**). The similar trends observed in females, patients aged ≥65 years, low BMIs, early TNM stages (I/II), history of drinking, surgery, and chemoradiotherapy had no statistical significance (P>0.05).

**Table 3:** Inflammation-water score.

Inflammation-water score	Score
NLR <2.42 and ICW/TBW ratio ≥0.61	0
NLR ≥2.42 or ICW/TBW ratio <0.61	1
NLR ≥2.42 and ICW/TBW ratio <0.61	2

NLR, neutrophil-to-lymphocyte ratio; ICW/TBW ratio, intracellular water/total body water ratio.

**Table 4:** Cox regression analysis of Inflammation-water score associated with overall survival.

Characteristics	N (cases)	Model a		Model b		Model c	
		HR (95% CI)	*P*	HR (95% CI)	*P*	HR (95% CI)	*P*
IWS = 0	205 (34)	ref.		ref.		ref.	
IWS = 1	297 (77)	1.51 (1.01, 2.27)	0.044	1.51 (1, 2.27)	0.049	1.49 (0.98, 2.24)	0.06
IWS = 2	126 (60)	3.37 (2.21, 5.14)	<0.001	3.11 (1.97, 4.92)	<0.001	2.86 (1.77, 4.63)	<0.001
p for trend	628 (171)		<0.001		<0.001		<0.001
Categories							
IWS = 0	205 (34)	ref.		ref.		ref.	
IWS ≥1	423 (137)	1.99 (1.37, 2.9)	<0.001	1.84 (1.25, 2.71)	0.002	1.74 (1.17, 2.57)	0.006

IWS, inflammation-water score.

Model a: Not adjusted.

Model b: Adjusted for age, sex, BMI, and TNM stage.

Model c: Adjusted for age, sex, BMI, TNM stage, smoking, drinking, surgery, and chemoradiotherapy.

### Comparison of the predictive abilities of NLR, ICW/TBW ratio, and IWS

The predictive abilities of the NLR (<2.42 and ≥2.42), ICW/TBW ratio (<0.61 and ≥0.61), and IWS were compared in **Table S3**. In the C-index model, the predictive ability of the IWS (C-statistic: 0.621) was higher than that of the NLR (C-statistic: 0.584) and ICW/TBW ratio (C-statistic: 0.582). In the cNRI and IDI index models, the predictive ability of the NLR was significantly lower than that of the IWS (difference: −0.291 and 95% CI: −0.418~−0.129; difference: −0.056 and 95% CI: −0.099~−0.011, respectively). The predictive ability of the ICW/TBW ratio was significantly lower than that of the IWS (difference −0.195, 95% CI −0.336~−0.024) in the cNRI model.

### Association between the ICW/TBW ratio and nutrients and muscle

We found that patients with lower ICW/TBW also had lower total protein, albumin, serum creatinine, and albumin/globulin ratio compared with patients with higher ICW/TBW (**Figure S6**). As shown in **Figure S7**, we found that patients with low ICW/TBW also had low FFM and KPS.

## Discussion

To the best of our knowledge, this study was the first to investigate the association between systemic inflammatory markers and water components and OS in CRC. In this study, we found that a high NLR and a low ICW/TBW ratio were independent prognostic indicators of poor survival (NLR and ICW/TBW ratio were mutually adjusted), and the NLR had no linear correlation with the ICW/TBW ratio. Meanwhile, we constructed an IWS based on the ICW/TBW ratio and NLR. The IWS had a better predictive ability than the NLR and ICW/TBW ratio. Compared to patients with an IWS of 0, those with an IWS of ≥1 had a poorer OS in CRC. In the stratified analysis, we found that the IWS more suitable as a prognostic indicator in advanced cancer (TNM stages III and IV), which has a greater burden of cancer-associated inflammation and excessive nutrient consumption, leading to an increased inflammatory marker (NLR) and a decreased nutrient-related marker (ICW/TBW ratio).

The negative association between NLR and OS in patients with cancer has been widely recognized as a reliable indicator of systemic inflammation (16, 17), especially in patients with advanced cancer, which is in agreement with previous studies (17–19). The mechanism underlying the association between a high NLR and poor prognosis in patients with cancer is unclear; a potential mechanism may be the relation of a high NLR to increased neutrophils and decreased lymphocytes. Chronic inflammation can promote the dedifferentiation and proliferation of tumor cells, promoting tumor progression (2, 20). Many studies have shown that neutrophils can directly or indirectly promote tumor cell growth and metastasis by regulating the tumor microenvironment. In addition, an increased serum neutrophil concentration has a negative effect on the cytotoxicity of natural killer cells and lymphocytes, thereby inhibiting the antitumor immune response (21, 22). In tumors, inflammatory infiltration composed of lymphocytes act as an immunosurveillance mechanism, inducing the production of anti-tumor-associated cytokines, such as *IFN-γ*, and inhibiting tumor proliferation (23).

Previous studies have shown that intracellular dehydration may occur in patients with cancer without edema (10), critically ill patients (13), and hemodialysis patients (24). Intracellular dehydration is typically related to an increased extracellular osmotic pressure. In cancer-related cell damage and extracellular hyperosmosis, intracellular fluid flows out of the cell. Intracellular dehydration may cause decreased various synthesis reactions in cells, hinder protein synthesis, affect protein structure and function, and eventually lead to the destruction of cell structure and function (25). Therefore, a decreased nutrient synthesis caused by a decreased ICW/TBW ratio may leave the patients in a state of malnutrition. Furthermore, preliminary studies have shown that intracellular dehydration may lead to decreased muscle quantity accompanied by decreased muscle strength and impaired function (26). In older adults, muscle strength seems to be related to muscle quality rather than quantity (25). Muscle strength is the primary determinant of muscle function. Cell atrophy and damage caused by the transfer of water components will lead not only to a decline in skeletal muscle quality and function but also poor quality of life, which have significant adverse consequences for survival and prognosis. These may explain why the all-cause mortality of CRC significantly increased with a decrease in the ICW/TBW ratio in our study.

Water composition is an indicator of skeletal muscle quality; the relationship between the skeletal muscle and inflammation has been widely discussed. Controlling inflammation is one of the body’s protective mechanisms; however, inflammation in cancer is not self-limited, so damage repair procedures are constantly activated, leading to chronic inflammation (27); a long-term inflammatory state induces chronic cell damage, further aggravating cell dehydration. The phenotypic imbalance of macrophages observed in chronic inflammation is associated with the impaired function of major cells needed for skeletal muscle regeneration, which results in the abnormal accumulation of profibrotic factors and delayed activation of satellite cells, hindering the repair and regeneration of skeletal muscles (28). Additionally, chronic inflammation can lead to a sustained decrease in skeletal muscle sensitivity, which is continuously reduced by inadequate nutrient acquisition in cancer. Since the water content of the skeletal muscle is relatively high, loss of the skeletal muscle mass leads to decreased ICW. Impaired nutrient synthesis due to intracellular dehydration can lead to inadequate nutrient acquisition in the skeletal muscle. Cancer-related cell impairment and inflammation lead to the transfer of the intracellular fluid to the extracellular surface, and the decrease in the TBW may not be as evident as that of the ICW. Therefore, a decreased ICW/TBW ratio may indicate a decline in skeletal muscle mass and function in patients with cancer. Inflammation can also directly reduce skeletal muscle mass and hinder muscle repair and nutrition uptake. Therefore, the combination of inflammation and water composition can better reflect the extent of skeletal muscle injury, which is conducive to the evaluation of systemic inflammation and nutritional status of the patients as well. Assuming that the low ICW/TBW ratio in this study represented a reduced skeletal muscle mass, the result is consistent with that of a previous study (29) stating that the risk of death in patients with CRC doubles when inflammation is combined with muscle loss, and the combination of the two has a better prognostic stratification effect than a single indicator.

Our study has some limitations. First, the small sample size may lead to biased statistical analysis. Second, the difference in muscle mass between BIA and CT scans could not be compared due to the lack of CT scan muscle mass. Finally, this study was unable to elucidate the mechanistic network between inflammation, water composition, and poor OS in CRC patients. More research is needed to explain the mechanisms by which inflammation and cellular dehydration reduce survival to provide information to guide treatment decisions.

A high NLR and a low ICW/TBW ratio were independent risk factors for poor prognosis in patients with CRC. The combination of the two can provide a better prognostic prediction effect.

## Data availability statement

The original contributions presented in the study are included in the article/**Supplementary Material**. Further inquiries can be directed to the corresponding author.

## Author contributions

H-PS, S-QL, H-LX, Y-ZG, and G-TR contributed to the acquisition and analysis of the data. QZ, M-MS, H-YZ, X-RL, MT and XZ contributed to the analysis of the data. XS, C-HS and WL contributed to the acquisition, analysis, and interpretation of the data. All authors contributed to the article and approved the submitted version.

## Funding

This study was supported by the National Key Research and Development Program to H-PS (No. 2017YFC1309200) and the Beijing Municipal Science and Technology Commission (SCW2018-06).

## Acknowledgments

We would like to express our sincere thanks to the INSCOC project members for their substantial work on data collection and patient follow-up.

## Conflict of interest

The authors declare that the research was conducted in the absence of any commercial or financial relationships that could be construed as a potential conflict of interest.

## Publisher’s note

All claims expressed in this article are solely those of the authors and do not necessarily represent those of their affiliated organizations, or those of the publisher, the editors and the reviewers. Any product that may be evaluated in this article, or claim that may be made by its manufacturer, is not guaranteed or endorsed by the publisher.

## Abbreviations

CRC, colorectal caner; BMI, body mass index; ICW, intracellular water; TBW, total body water; ICW/TBW ratio, intracellular water/total body water ratio; FFM, fat free mass; FM, fat mass; NLR, neutrophil-to-lymphocyte ratio; OS, overall survival; C-index, concordance index; IDI, integrated discriminant improvement; cNRI, continuous net reclassification index.
