# Supplementary material for: Association between systemic inflammation and water composition and survival in colorectal cancer
Source: Front Oncol. 2022 Oct 24;12:896160. doi: 10.3389/fonc.2022.896160 (PMC9638509; doi:10.3389/fonc.2022.896160)
Supplement: Supplementary file 1 [file DataSheet_1.pdf]

Table S1. Baseline characteristics of colorectal cancer patients stratified by IWS.

Characteristics	IWS			p
	0<br>N=205	1<br>N=297	2<br>N=126	
Sex, male, n (%)	125 (60.98)	176 (59.26)	75 (59.52)	0.925
Age (median [IQR])	56.00 [12.00]	60.00 [14.00]	65.00 [12.00]	<0.001
BMI (mean (SD))	23.05 [4.89]	22.89 [4.58]	22.23 [4.20]	0.015
Smoking, yes, n (%)	76 (37.07)	117 (39.39)	46 (36.51)	0.803
Drinking, yes, n (%)	44 (21.46)	61 (20.54)	21 (16.67)	0.549
Surgery, yes, n (%)	27 (13.17)	83 (27.95)	57 (45.24)	<0.001
Chemoradiotherapy, yes, n (%)	145 (70.73)	186 (62.63)	48 (38.10)	<0.001
Tumor stage, n (%)				0.394
I	10 (4.88)	18 (6.06)	8 (6.35)	
II	43 (20.98)	66 (22.22)	24 (19.05)	
III	91 (44.39)	123 (41.41)	43 (34.13)	
IV	61 (29.76)	90 (30.30)	51 (40.48)	
Neutrophil (median [IQR])	2.48 [1.37]	3.47 [2.78]	5.10 [3.83]	<0.001
Lymphocyte (median [IQR])	1.74 [0.68]	1.39 [0.71]	1.25 [0.76]	<0.001
ICW (median [IQR])	22.00 [6.40]	21.60 [6.50]	20.20 [5.83]	<0.001
TBW (median [IQR])	35.70 [10.40]	35.60 [10.40]	33.60 [9.33]	0.018
NLR (median [IQR])	1.45 [0.74]	2.63 [2.84]	4.23 [3.23]	<0.001
ICW/TBW (median [IQR])	0.62 [0.01]	0.61 [0.01]	0.60 [0.01]	<0.001
Status, death, n (%)	34 (16.59)	77 (25.93)	60 (47.62)	<0.001

Notes: BMI, body mass index; ICW, intracellular water; TBW, total body water; ICW/TBW ratio, intracellular water/total body water ratio; NLR, neutrophil-to-lymphocyte ratio.

Table S2. Cox regression analysis of the NLR and ICW/TBW ratio associated with overall survival.

Characteristics	Model a		Model b		Model c	
	HR (95%CI)	p	HR (95%CI)	p	HR (95%CI)	p
NLR						
Continuous (per SD)	1.14 (1.01,1.28)	0.030	1.17 (1.05,1.31)	0.004	1.12 (0.99,1.27)	0.065
by cut-off						
D1	ref.		ref.		ref.	
D2	1.92 (1.4,2.62)	<0.001	2.13 (1.54,2.95)	<0.001	2.00 (1.42,2.81)	<0.001
Quartiles						
Q1	ref.		ref.		ref.	
Q2	0.91 (0.55,1.52)	0.731	0.84 (0.50,1.41)	0.506	0.80 (0.47,1.35)	0.402
Q3	1.50 (0.96,2.35)	0.073	1.53 (0.96,2.43)	0.074	1.44 (0.90,2.30)	0.132
Q4	1.76 (1.14,2.71)	0.011	2.06 (1.32,3.21)	0.001	1.83 (1.14,2.94)	0.012
p for trend		0.002		<0.001		0.001
ICW/TBW						
Continuous (per SD)	0.74 (0.63,0.87)	<0.001	0.80 (0.66,0.96)	0.018	0.82 (0.68,0.99)	0.040
by cut-off						

D2	ref.		ref.		ref.	
D1	1.86 (1.37,2.51)	<0.001	1.44 (1.04,1.99)	0.027	1.40 (1.01,1.95)	0.042
Quartiles						
Q4	ref.		ref.		ref.	
Q3	0.94 (0.58,1.53)	0.818	0.90 (0.55,1.47)	0.667	0.96 (0.58,1.58)	0.870
Q2	1.68 (1.09,2.59)	0.019	1.32 (0.82,2.11)	0.250	1.42 (0.88,2.29)	0.149
Q1	2.04 (1.33,3.12)	0.001	1.53 (0.94,2.47)	0.085	1.48 (0.91,2.41)	0.110
p for trend		<0.001		0.032		0.048

Notes: NLR, neutrophil-to-lymphocyte ratio; ICW/TBW, intracellular water/total body water ratio

Model a: No adjusted.

Model b: Adjusted for age, sex, BMI, TNM stage.

Model c: Adjusted for age, sex, BMI, TNM stage, smoking, drinking, surgery, chemoradiotherapy.

Table S3. Comparative analysis of the discrimination of NLR, ICW/TBW ratio, Inflammation-water score for all-cause mortality in patients with cancer.

Discrimination Ability	C-statistic	cNRI		IDI	
		Difference	p	Difference	p
NLR	0.584(0.545,0.624)	-0.291(-0.418, -0.129)	0.012	-0.056(-0.099, -0.011)	0.020
ICW/TBW	0.582(0.542,0.621)	-0.195(-0.336, -0.024)	0.030	-0.038(-0.080, 0.007)	0.094
IWS	0.621(0.576,0.665)	ref.		ref.	

Notes: cNRI, continuous net reclassification improvement; IDI, integrated discrimination improvement; NLR, neutrophil-to-lymphocyte ratio; ICW/TBW, intracellular water/total body water ratio; IWS, inflammation-water ratio.

Figure S1. Flow chart.

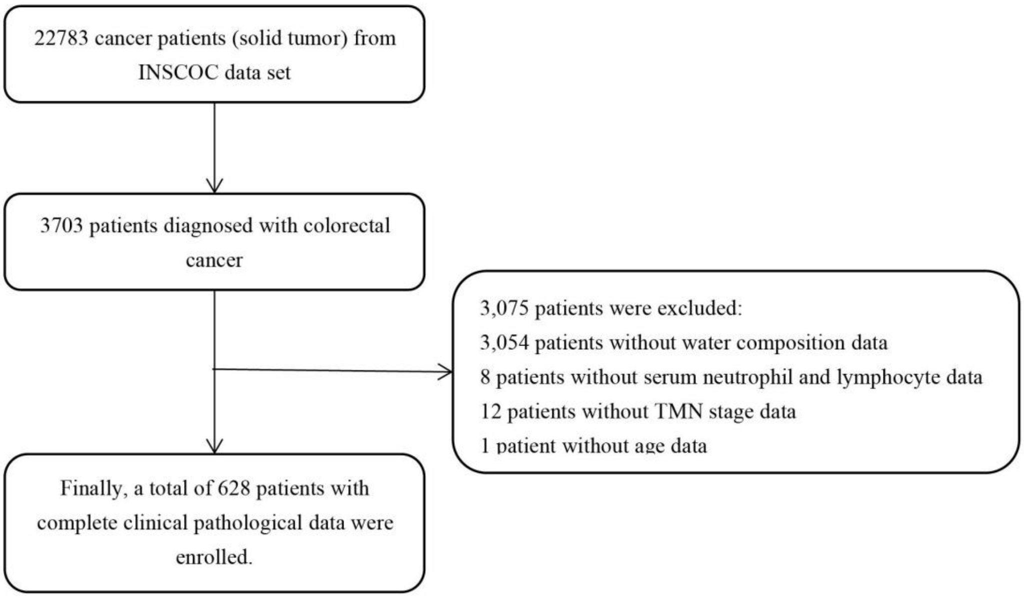

Figure S2. A comparison of the predictive capabilities of the ICW/TBW ratio, FFM, and the FM.

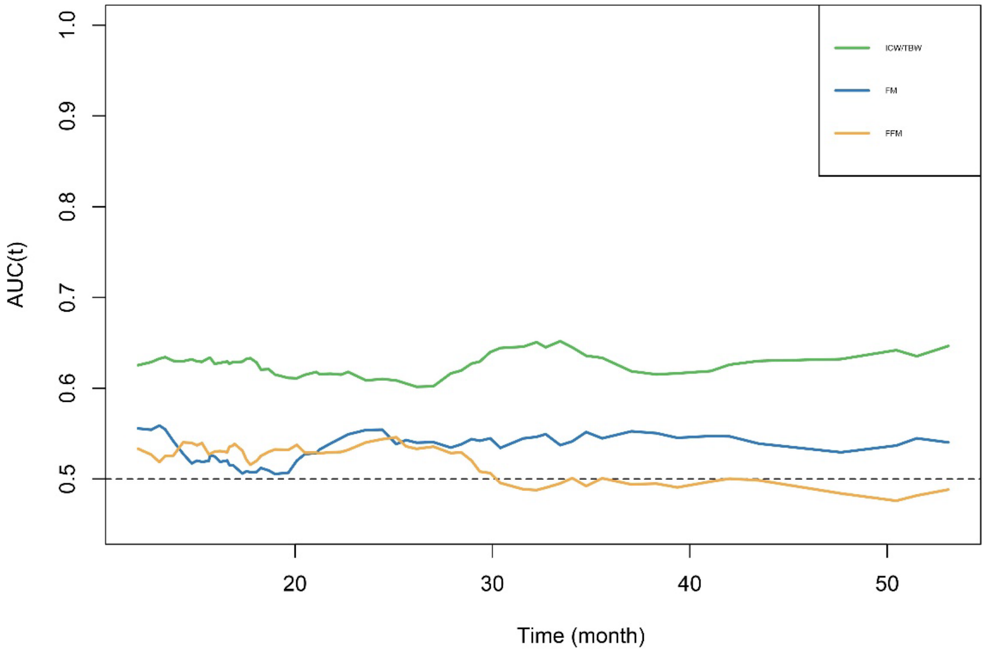

ICW/TBW ratio: intracellular water/total body water ratio; FM: fat mass; FFM: fat free mass

Figure S3. Linear relationship between the NLR and ICW/TBW ratio

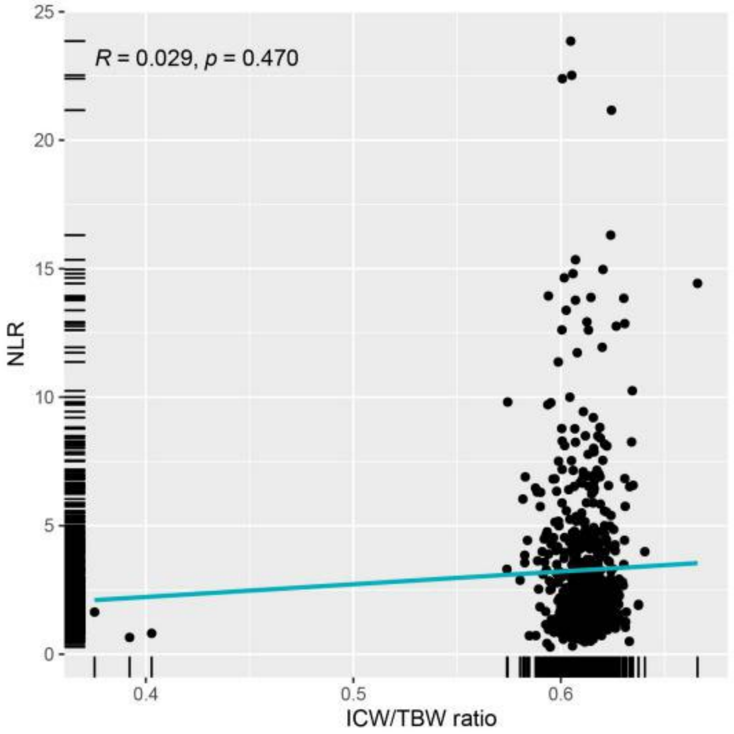

NLR: neutrophil-lymphocyte ratio; ICW/TBW ratio: intracellular water/total body water ratio

Figure S4. Stratified survival analysis of the NLR, ICW/TBW ratio and inflammation-water score based on TNM stage

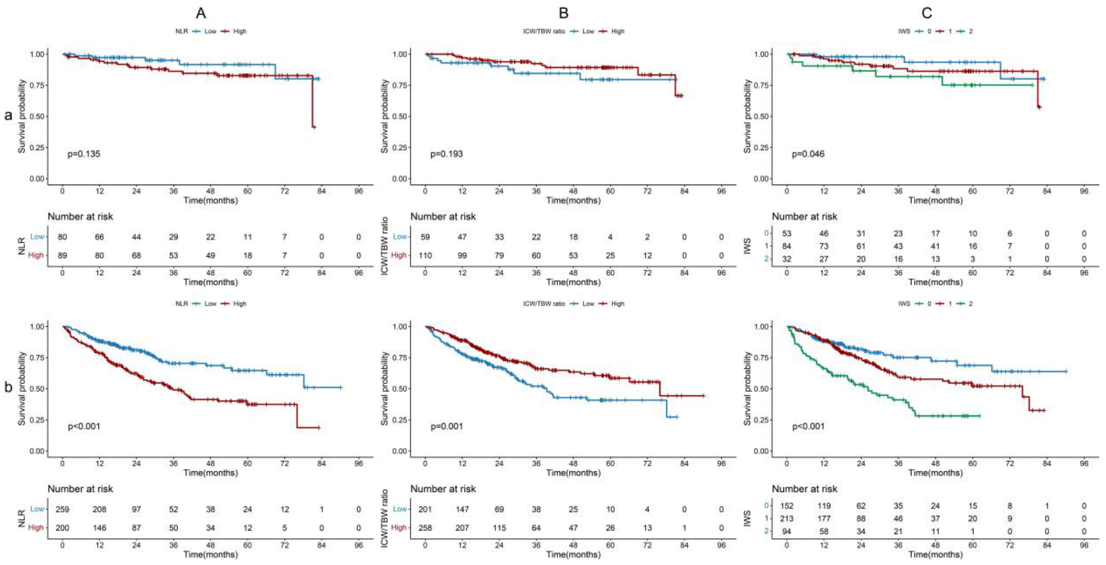

A- NLR: neutrophil-lymphocyte ratio; B- ICW/TBW ratio: intracellular water/total body water ratio; C- IWS: inflammation-water ratio

Figure S5. Association between the NLR and ICW/TBW ratio and hazard risk of overall survival in various subgroups

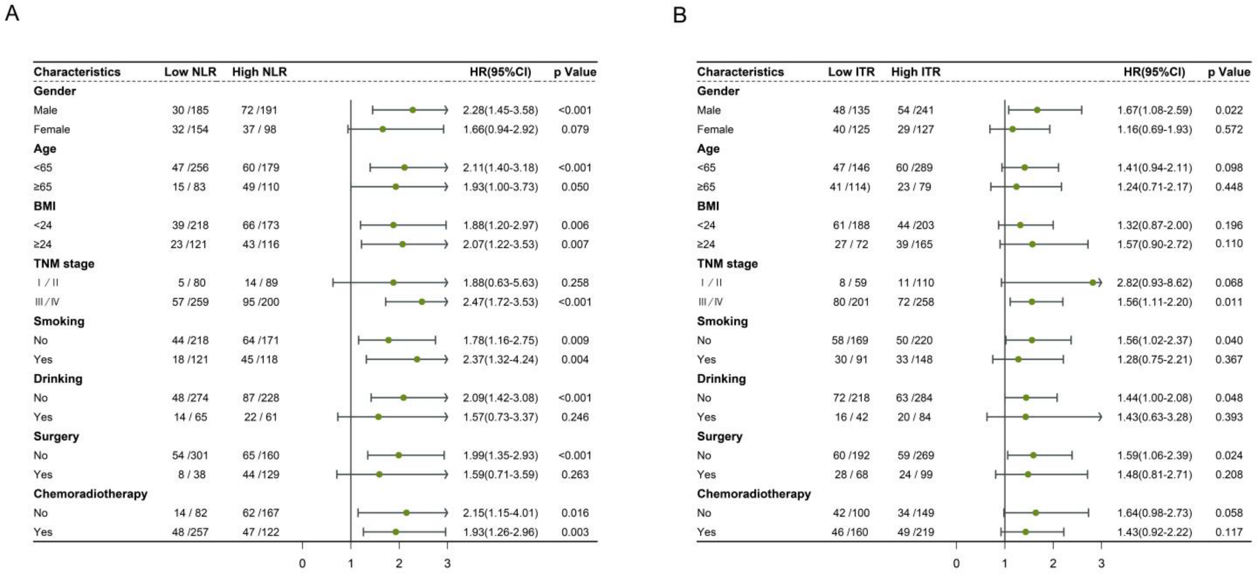

A- NLR: neutrophil-lymphocyte ratio; B- ICW/TBW: intracellular water/total body water ratio
Adjusted for age, sex, BMI, TNM stage, smoking, drinking, surgery, and chemoradiotherapy

Figure S6. Nutrients in different population stratified by ICW/TBW.

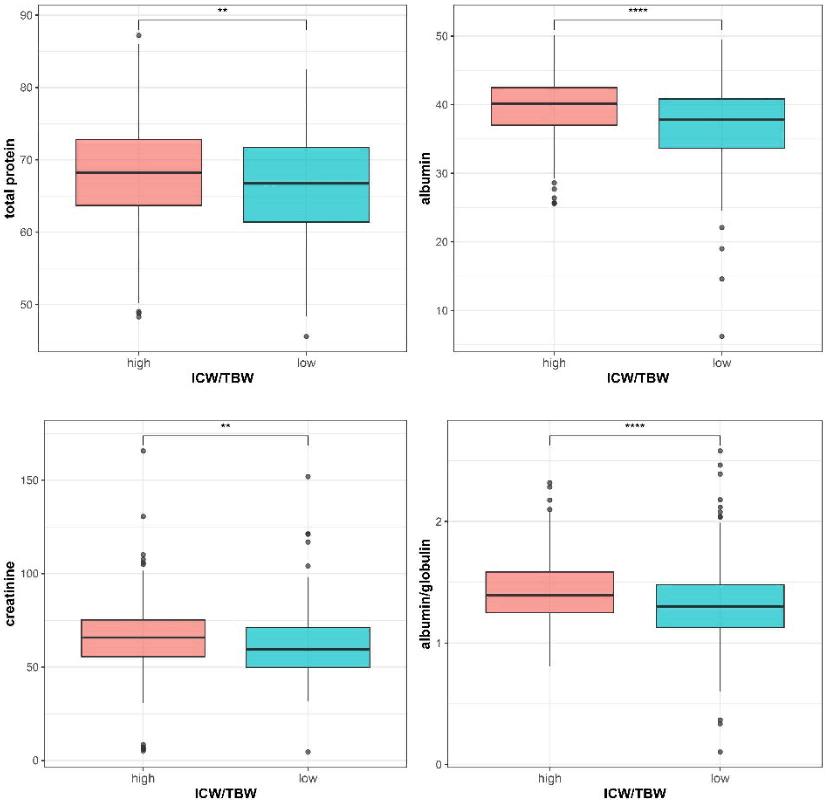

ICW/TBW ratio: intracellular water/total body water ratio

Figure S7. Nutrients in different population stratified by ICW/TBW and sex.

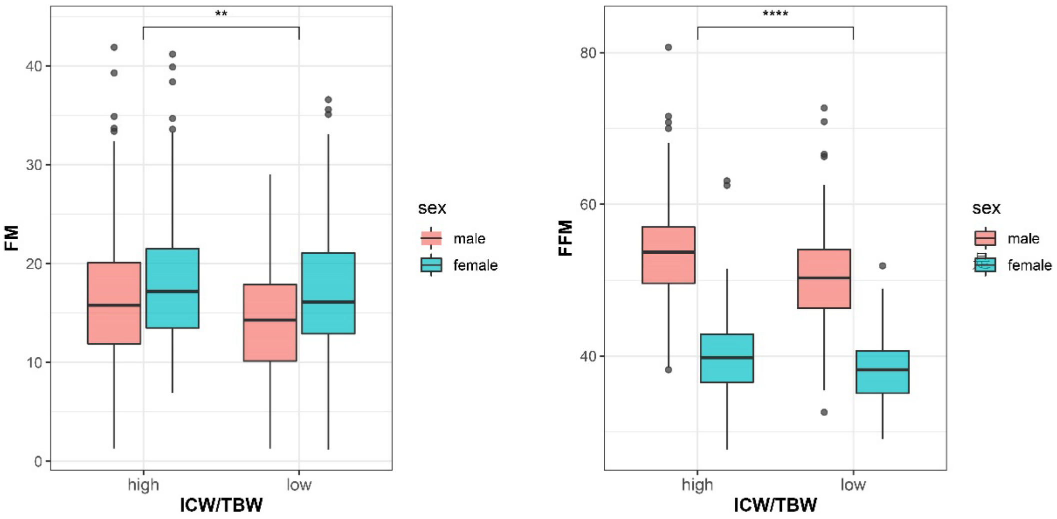

ICW/TBW ratio: intracellular water/total body water ratio; FM: fat mass; FFM: fat free mass
